# Microparticle Size and Quantities Effect on the Mechanical Features of End of Life Tires in Thermoplastic Composites

**DOI:** 10.3390/ma13235561

**Published:** 2020-12-06

**Authors:** Marc Marín-Genescà, Jordi García-Amorós, Ramon Mujal-Rosas, Lluís Massagués Vidal, Xavier Colom Fajula

**Affiliations:** 1 Mechanical Engineering Department, ETSEQ-URV, Països Catalans, 26, 43007 Tarragona, Spain; 2 Electrical Engineering Department, ETSE-URV, Països Catalans, 26, 43007 Tarragona, Spain; jordi.garcia-amoros@urv.cat (J.G.-A.); lluis.massagues@urv.cat (L.M.V.); 3 Electrical Engineering Department, EET-UPC, Colom, 1 08222 Terrassa, Spain; mujal@ee.upc.edu; 4 Chemical Engineering Department, EET-UPC, Colom, 1 08222 Terrassa, Spain; Xavier.colom@upc.edu

**Keywords:** GTR, recycling, reuse, mechanical properties, composites, materials

## Abstract

Currently, the huge use of tires generates large quantities of waste material which represents a severe environmental problem. The common technique used for processing waste tires is crushing using mechanical methods and separating tire components like fibers, metals, and rubber from the used tire. The aim of this research is the recycling of this rubber from crushed tires, called ground tire rubber (GTR). With this aim, the manuscript analyses key mechanical properties of the thermoplastic composites produced by blending of crushed and micronized small particles of waste rubber tires with several industrial thermoplastic polymers. These types of composites are defined based on the total amount GTR in percent by weight, in the composite, and also, the particle sizes used in each case, so these aforementioned two variables (microparticle size and amounts) along with seven common industrial polymers define a series of composites for which the mechanical properties were tested, studied, analyzed and finally presented. Finally, the results obtained show that this proposed recycling method could be a way to enhance some specific polymer properties and could contribute to reducing the total of end of life used tire stocks environmental problem.

## 1. Introduction

The environmental problem of the great worldwide stock of out of end of use tires [1,2,3] has focused the efforts of the governments, companies and all of society to search for solutions for recycling these used tires. Essentially, a thermoplastic polymer act as a polymeric array and the micronized elastomeric part acts as a dispersed mixed material [4,5,6]. In many two-phase polymeric blends, like the composites analyzed in some research works [7,8], the interfacial compatibility between both phases is a key issue for achieving acceptable mechanical properties. In recycled or reused elastomers, like the analyzed case of ground tire rubber (GTR), the initially predicted compatibility between both phases is small. A way to increase the affinity between both components is to lower the degree of cross-linking of GTR by devulcanization methods, which improves the interfacial adhesion and thus the mechanical characteristics [9,10,11]. Important transformations in features are also seen when the diameter of the coating particles is modified [12]. The use of these GTR particles as a mixer component in composite materials has been studied in many works on behavior characterization of thermoplastic polymers with GTR reinforcements, analyzing different composites, but never specifically in a mechanical study-analysis and comparison of properties [13,14,15,16,17,18,19]. The presence of these out of use tire particles in polymer matrix composite materials modifies the mechanical behavior. The size of particles is restricted and was chosen based on a simple and cheap industrial recycling method to obtain the classification in the three selected particle diameters which are: <200 μm, 200 μm–500 μm, and finally >500 μm. Thus, the research aim was to determine what percentage of GTR can be added to seven different thermoplastic polymer matrices (PVC, EVA, HDPE, PP, PA, ABS, and PS) while keeping the polymer initial microstructure [20,21,22] within a suitable range of mechanical values. This could be a way to add GTR, which is difficult to recycle waste, to various industrial processes. To this end, we have analyzed some concentrations of polymer/GTR (from 0–70% of GTR concentrations particles by weight), with three-particle diameters. The GTR constitutes the reinforcing agent here. Tires can contain some important amounts of carbon black (CB), in this sense, some authors [23] have shown that carbon black when used as a reinforcement in composite materials, increases the mechanical characteristics. Thermoplastic composites can be heterogeneous, and their properties depend, among others, on different factors such as the quantity, diameter, shape, and compatibility of the added phase. Saad et al. [24] tested different samples of PVC containing variable proportions of carbon black additives (CB), showing that PVC with CB produces composites with good mechanical properties. Summarizing, our research aims were to study and compare the mechanical behavior of some composite materials obtained by mixing different polymers with different amounts of GTR (up to 70%), to check their response in function of the amount and particle size of micronized elastomers (GTR). Therefore, the aim of this research was the analysis the mechanical behavior of waste composite materials to use some of these composites for different applications, and in general, to produce an output to reusable materials for new applications. In this sense, the GTR could not be used in high requirements application where polymers are already used, but these mixtures could provide a partial solution of the difficult recycling of these materials.

## 2. Methodology

### 2.1. Thermoplastic Polymers

Seven thermoplastic polymers were used in this comparative study: high-density polyethylene (HDPE); polyvinyl chloride (PVC); ethylene vinyl acetate (EVA) copolymer, with the composition 18% of vinyl acetate and 82% ethylene; polypropylene (PP); ABS, composed by 20% of butadiene, 30% of acrylonitrile, and finally 50% of styrene; polyamide 6 (PA), which is a semi-crystalline material and polystyrene (PS) styrene-butadiene-styrene, which is an amorphous thermoplastic. All seven of these polymers are widely used in industry and in many applications, polymer’s technical data are provided in Table 1. The end of life tires (GTR), with a microparticle size lower than 700 μm, have been tested by thermogravimetry (TGA) which confirmed that the CB content was nearly 35% by weight. Finally, the micronized GTR was distributed by a sieve in three different categories in function of diameter particle size: less than 200 μm, from 200 to 500 μm, and finally higher than 500 μm. GTR particles added into the polymeric matrix of the composite, have not received any pre-treatment and have been crushed and separated by size but have not been devulcanized nor treated with other additives, and have been mixed in a process of mechanical mixing with a laminating machine (mixer machine, Brabender, Duisburg, Germany), to finally obtain the different composites. Five specimens were tested from each percent amount of GTR (polymers/GTR composites), so each value obtained from this research is the average result from five repetitions. Regarding the current deviation or deviation from the mean, we have rejected the obtained values over than deviation away from the average value in each tested composite; the average errors founded are in the range between 7.1% and 2.2%.

### 2.2. Composite Processing

Once separated into the three different sizes, the GTR particles, the recycled tire particles were dried at 100 °C for 24 h. Five specimens of each composite (thermoplastic/GTR), changing the particle tires amount in each case (5%, 10%, 20%, 40%, 50%, and 70% of GTR by weight), were mixed for each diameter of particle (<200 μm, 200 μm–500 μm, and finally >500 μm). The mixing process was performed on a Brabender plasticizer machine (Brabender, Duisburg, Germany), at different temperatures (Table 2). The rotational rollers’ speed was 100 revolutions per minute. Blending time was between 8 and 10 min with 2 or 3 min of preliminary treatment depending on matrix and 6–7 min of mixing with different amounts of GTR. Composite sheets were obtained using a hot plate press machine for 10 min at pressure fixed at 200 bar and using different pressing temperatures depending on the used polymer (Table 2) for 10 min. Specimens for testing were set up according to ASTM-D-638 type V standard, also a specimen pure polymer (0% GTR), in each case, was manufactured with the method to obtain results to compare, and to perform the comparative study-analysis.

### 2.3. Mechanical Test

Mechanical type test: stress-strain tests were performed using an Instron 3366–10 kN machine stress-strain tester (Instron, Norwood, MA, USA), following the ASTM-D-638 standard. Some relevant variables are the test speed (20 mm/min), and the test environment variables were the following: test temperature, 23 ± 2 °C, and relative humidity, 50%. The mechanical characteristics obtained according to the GTR amounts in the polymeric matrix and the three different particle sizes include key mechanical properties: tensile stress, Young modulus, toughness, and elongation. Five samples for testing were used in each case. Statistical variables were obtained like, the mean and standard deviation for all the features, leaving out the test specimens that showed defects.

## 3. Results and Mechanical Properties

The results by the stress-strain tests of the different polymeric composites that have been analyzed with some GTR amounts and the three particle diameters define the thermoplastic polymer matrix composites. 0% of GTR, in the different figures, corresponding to the neat polymer, so in all the figures it corresponds to 0% GTR to the neat polymer tested in each case. The figures below show the mechanical properties in function of the percentage of GTR contents and the particle diameter.

### 3.1. Mechanical Properties of PVC/GTR Composites

**Figure 1:**
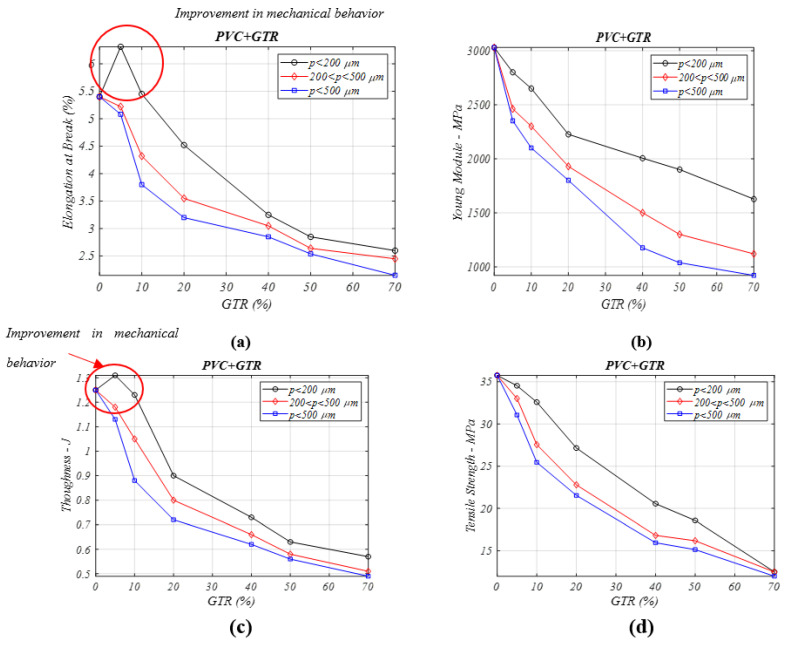
Mechanical properties analysis for different percentages of PVC/GTR composites and particle diameters: (**a**) elongation at break (%), (**b**) Young’s modulus (MPa), (**c**) Toughness (J) (**d**) tensile stress (MPa).

Figure 1a shows the elongation at break property analyzed in the PVC + GTR composites. The lower GTR microparticle size (*p* < 200 μm) material shows optimal behavior, and for small concentrations (5%), the elasticity is higher than in neat PVC with no reinforcement. As the GTR percentage increases, for the same particle diameter, the values decrease with 10% of GTR. This decrease is higher for 20% amounts, and levels out for 40–50–70% of GTR. The 200–500 μm, and >500 μm particle sizes and rises in the GTR amount in the PVC matrix always produce reductions in elongation, due to the poor interfacial adhesion that these huge GTR amounts cause in the PVC composite matrix.

Figure 1b illustrates the Young’s modulus of the composite (PVC+GtTR), where a decrease in Young‘s modulus with GTR filler addition is seen, as the rigidity resulting from increasing amounts GTR (>10%) decreases compared to neat PVC: 2800 MPa, for 5% GTR, 1627 MPa, for 70% GTR, and in lowest particle diameters (<200 μm). It can be seen how the Young’s modulus decreases for the largest particle diameters, from a Young’s modulus of 2461 MPa in 5% GTR composites, to 1120 MPa for 70% GTR in polymeric composites, for 200–500 μm GTR particle diameters, and to 921 MPa for a high microparticle size (>500 μm), for equal GTR amounts. This behavior is caused by the fact larger particles have lower interfacial adhesion and thus a high probability of experiencing cracks; another consideration is the accumulation of particles during the composite production process. While the GTR content increases in the composites, the interfacial adhesion is worsening and this causes a decrease of stiffness in every analyzed case, so for 40–50% of GTR, the values are 2005 MPa–1900 MPa, for <200 μm diameter particles (Figure 1b).

Figure 1c shows how the toughness property behavior changes significantly for different GTR particle diameters, since, for smaller particles (<200 μm) and the lowest GTR amounts (5%), the composites show good interfacial adhesion and a clear improvement in this specific property, whereas for 200–500 μm, and >500 μm particle composites result in worsening toughness properties, with a decrease in the breakage energy, also seen with with lowest GTR amount (5% GTR).

In Figure 1d the tensile strength is analyzed. For low GTR percentages (5% and 10%) and the smallest particle diameters <200 μm), the strength value decreases slightly, whereas for higher concentrations of GTR (>20%) the values show a decrease, which is explained by the low affinity between both components, and worse interfacial adhesion as the amount of reinforcement is rising, and this trend falls away for higher particle diameters (>200 μm).

The frailness in the composites (PVC + GTR) resulting from adding GTR particles as reinforcement worsens most of the mechanical properties from the outset analyzed. The maximum GTR amounts in the polymeric matrix show the poor compatibility between the components, which causes poor mechanical behavior in all circumstances, for the two particle sizes analyzed.

### 3.2. Mechanical Properties of EVA/GTR Composites

**Figure 2:**
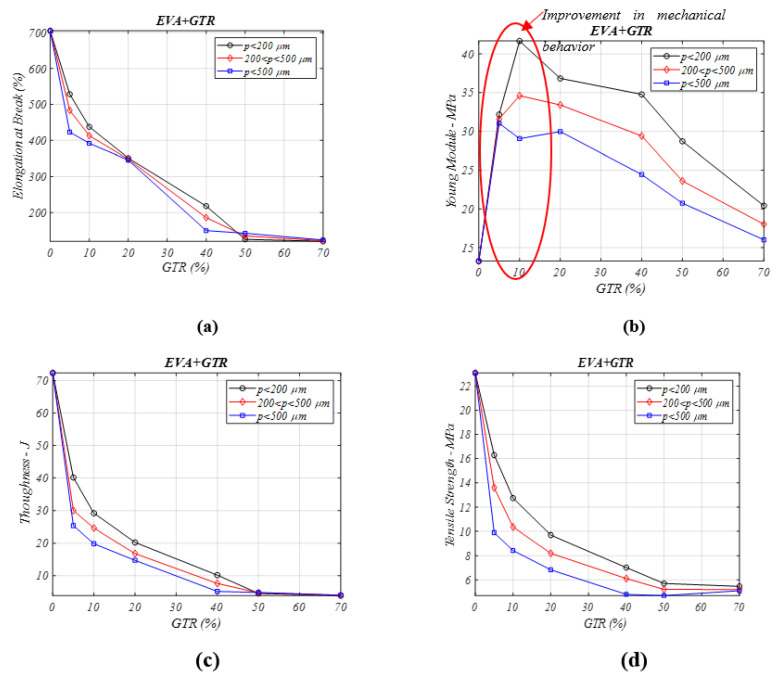
Mechanical properties analysis for different percentage of EVA/GTR composites and particle diameters: (**a**) elongation at break (%), (**b**) Young’s modulus (MPa), (**c**) toughness (J) (**d**) tensile stress (MPa).

The coalescence of end of used tire amounts in polymeric EVA composites produces a contraction in the elongation (Figure 2a) and toughness (Figure 2c) features. For *p* < 200 μm, the elongation of the EVA/GTR [25] changes from 704% to 351%, for neat EVA and 20% of GTR. The drops in elongation are due to the low interfacial adhesion and poor compatibility of both components. The low adhesion between phases directly affects the decrease in elongation at break and, consequently, the decrease of tensile strength and toughness.

**Figure 3:**
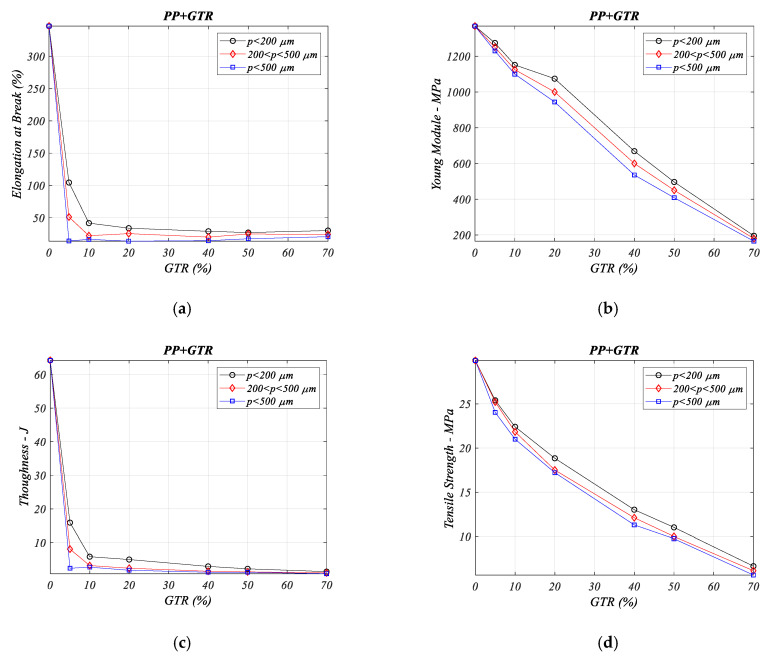
Mechanical properties analysis for some percentage of PP/GTR composites and particle sizes: (**a**) elongation at break (%), (**b**) Young’s modulus (MPa), (**c**) toughness (J) (**d**) tensile stress (MPa).

In Figure 2b, the composite’s Young’s modulus property is rising with the GTR amount (from 5% to 70%) regarding the neat EVA copolymer. The values analysis goes from a value of 13.2 to 41.7 MPa (higher value of Young modulus analyzed) for lower particle diameters *p* < 200 μm, and 5% GTR percent composite. The rigidity also increases for all particle sizes analyzed, from a neat polymer Young’s modulus value of 13.2→34.6 MPa the (*p* = 200–500 μm), and to 29 MPa (*p* > 500 μm) for the same amount of GTR (5%) in the composites. When the GTR percentage increases, the interfacial adhesion gets worse, and this damages the stiffness in all the analyzed samples. For the toughness property (Figure 3c), these drops are greater, and for particle sizes lower than 200 μm, the toughness property goes from 72.3 J (neat EVA, or EVA/0% GTR) → 29.2 J (EVA/10% GTR). The decrease is more accused for larger particle diameters and GTR percentage over 20%, so the optimum behavior is for particle sizes under of 200 μm. GTR addition decreases the tested mechanical features [26], with some exceptions, like for the Young’s modulus (Figure 2b).

Figure 2d shows the tensile-stress property We can see a contraction for lower GTR percentage, so, for GTR amounts in EVA composites of 5% or 10%, the decreases in tensile stress property compared to neat EVA are significant, from 23 MPa (neat EVA polymer)→16.2 MPa (5% GTR) and 12.7 MPa (10% GTR), for particle sizes lower than 200 µm. These drops are more meaningful for particle sizes larger than 200 μm. From 20% GTR percent in composites, the toughness falls steadily for all particle sizes and GTR amounts in the composites, which is caused by the poor compatibility between both phases when the amount of reinforcement is raised.

### 3.3. Mechanical Properties of PP/GTR Composites

The coalescence of end of life tire particles in the analyzed composites develops a major drop in Elongation (Figure 3a), the same for toughness property (Figure 3c). So, is seen that tenacity and elongation in polymeric composites with GTR major drop, regarding the neat PP polymer (in PP + 70% GTR: nearly 9 times contraction elongation property and 12 times drop values than relative hardness property). These decreases are caused by the imperfect adherence of the interface between both components in the composite formed by polypropylene and GTR.

Figure 3b reveals that for the Young’s modulus, the values decrease as more GTR filler is added to the PP matrix. For a small percent of GTR (from 5% to 20%), the decrease is slightly for lower that 200 μm particles. For larger GTR additions (from 40% to 70%) and *p* < 200 μm, the Young’s decreases modulus significantly with little difference according to the particle diameter used. Again, the behavior is caused by poor cohesion of the interface with the particles, which causes cracks and fractures in the interface that weaken the composites.

Figure 3d shows the tensile strength, where the tension drops are uniform and linear with decreasing values from neat PP polymer; differences between the particle sizes are not significant, (around 5% to 15%, comparing the extremes). The presence of GTR in the composites affects the interfacial adhesion decreasing in tensile strength in all studied PP/GTR composites, independently of the microparticle size. Again, agglomerations of GTR microparticles during the composite mixing fabrication process must be considered.

### 3.4. Mechanical Properties of HDPE/GTR Composites

**Figure 4:**
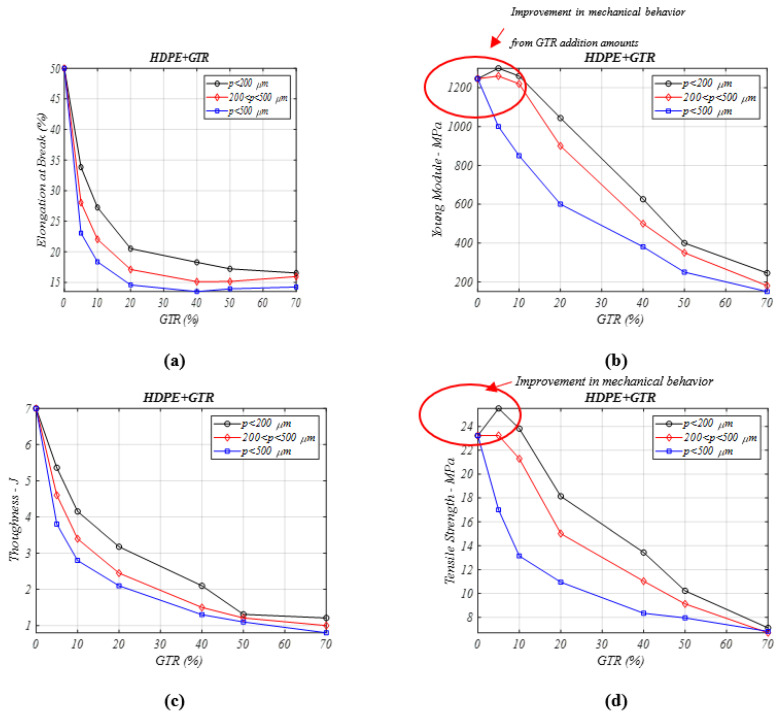
Mechanical properties analysis for different percentages of HDPE/GTR composites and particle diameters: (**a**) elongation at break (%), (**b**) Young’s modulus (MPa), (**c**) toughness (J) (**d**) tensile stress (MPa).

The coalescence of end-life tire particles in all PP+GTR samples caused a notable decrease of elongation (Figure 4a) in HDPE composites with GTR, the major drop being observed for the 5% GTR particles (GTR HDPE/GTR-5%) composites (50% to 34%), that decreases and increases, respectively, in the function of the particle diameter analyzed, and as we know, particle diameter is a key factor for the mechanical behavior in some composites [27]. Again, and like in other similar composites, drops in elongation have been verified [25,26], attributable to the low interfacial adhesion between the different parts in the analyzed and characterized composite samples.

**Figure 5:**
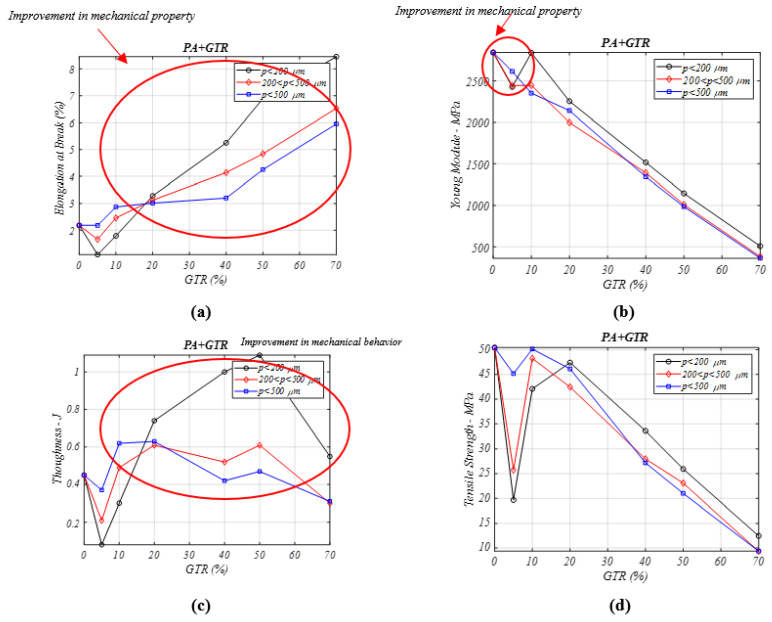
Mechanical properties analysis for different percentages of PA/GTR composites and particle diameters: (**a**) elongation at break (%), (**b**) Young’s modulus (MPa), (**c**) toughness (J) (**d**) tensile stress (MPa).

In Figure 4b the composite’s Young’s modulus is studied. For percentage > 10% GTR the rigidity is seen to increase compared to neat HDPE, with any reinforcement level (5–8%) of small-sized diameter particles (*p* < 200 μm) and has no changes are seen for particles between 200–500 μm. This fact is caused by the fact that composites with larger particles reduce the interactions between both components and this causes a low adhesion, which facilitates the propagation of fissures and cracks in the matrix interphase. This fact is obvious for particles over 500 µm, which show a remarkable drop in the analyzed mechanical properties. When the GTR amount increases, the interfacial compatibility diminishes and causes drops in the rigidity feature in all studied cases, independent of the microparticle diameter analyzed. For 40–70% of GTR, the values of the drop are 3–5 times lower than in neat analyzed HDPE polymer. In Figure 4c, the tensile strength for low GTR concentration amounts in the matrix (5–10%) and small microparticle sizes increases (from 4 to 8%), and for amounts over than 10%, this property shows a major drop, decreasing dramatically. Again, like many of the analyzed properties, the coalescence of out of use tire or GTR microparticles in composites of HDPE/GTR causes a remarkable value drop in the toughness (Figure 5d).

### 3.5. Mechanical Properties of PA/GTR Composites

The incorporation of end-of-life tire microparticles in PA composites causes a great rise in the elongation (Figure 5a) and toughness feature (Figure 5c). For GTR composites with microparticles (lower than 200 µm), these properties show an increase of values compared to neat PA samples analyzed [28]. The particle diameters affect the composite with an improvement for *p* > 500 μm and GTR amounts < 20%. The toughness property shows a similar behavior to the elongation at break property in PA + GTR composites. To summarize, neat PA, among the tested materials, has the lowest elongation-toughness properties, and logically these PA mechanical properties are improved by incorporation of GTR particles.

In Figure 5b the Young’s modulus of the PA/GTR composites is analyzed. Its value decreases as larger amounts of GTR is incorporated as a reinforcement, comparing to PA with no GTR. For low GTR concentrations (5% GTR), the stiffness remains similar to that of neat PA (2818 MPa (PA) vs. 2715 MPa (PA + 5% GTR), using particles of sizes <200 μm). Increasing the GTR percent (10–20% GTR), and for particle sizes lower than 200 μm, the decrease in Young’s modulus is still weak. Weak differences are noted between the results obtained as a function of particle diameter, which in this case is caused by the internal PA + GTR composites structure, which leaves open holes or spaces in the matrix, and these open spaces are readily filled by GTR particles for all three different sizes analyzed. Figure 5d analyzes the tensile strength property. It is mildly increased with 5% GTR, and decreases for percentages higher than 20% GTR. In conclusion, the compatibility between both components is good for minor GTR additions to PA composites. Particle size has a mild effect, as the differences are less than 10% for the extreme particle diameters.

### 3.6. Mechanical Property of ABS/GTR Composites

**Figure 6:**
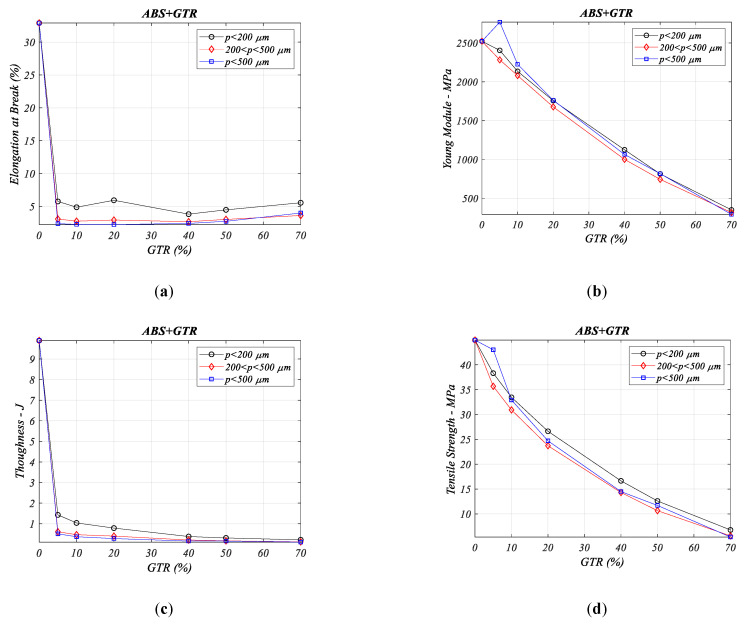
Mechanical properties analysis for different percentages of ABS/GTR composites and particle diameters: (**a**) elongation at break (%), (**b**) Young’s modulus (MPa), (**c**) toughness (J) (**d**) tensile stress (MPa).

In ABS copolymer and GTR composites (ABS + GTR) a decrease in all the analyzed properties is observed (see the Figure 6 analysis graphs) except for the Young’s modulus (Figure 6b) where the graph shows a small improvement for GTR/5% composites and in for larger particles >500 µm.

### 3.7. Mechanical Property of PS/GTR Composites

**Figure 7:**
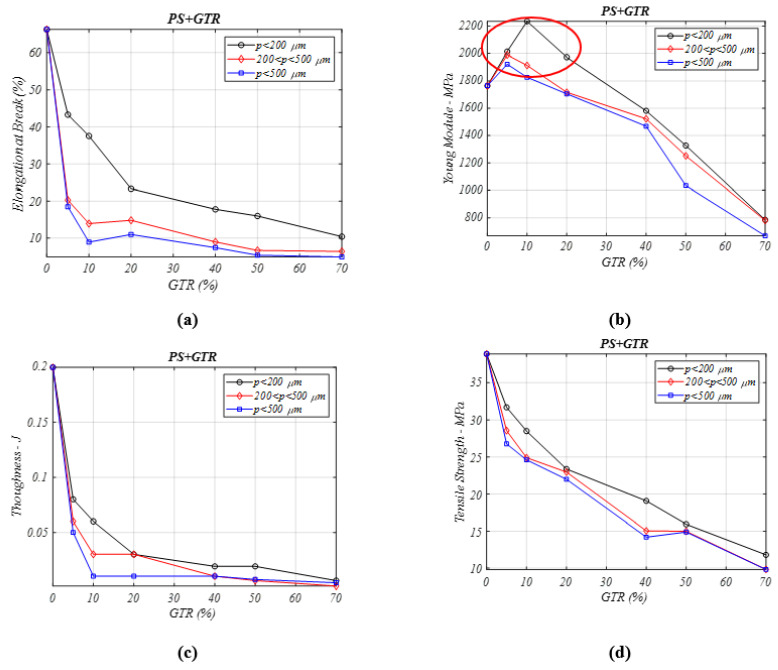
Mechanical properties analysis for different percentages of PS/GTR composites and particle diameters: (**a**) elongation at break (%), (**b**) Young’s modulus (MPa), (**c**) toughness (J) (**d**) tensile stress (MPa).

For all four mechanical properties analyzed in Figure 7, it can be seen that all the properties are deteriorating as the GTR amount is increased in the matrix. A remarkable exception is seen for the Young’s modulus (Figure 7a) in which the incorporation of elastomer particles (GTR) causes a rise in this property for the PS composite [29] for GTR percentages between 5% and 20%. This is another interesting fact that proves the importance of the diameter of the particles: The best Young’s modulus behavior is seen in composites with *p* < 200 µm. This difference may be caused by the PS internal structure that leaves open spaces could be occupied better by lower sized particles.

### 3.8. Stress-Strain Curves

**Figure 8:**
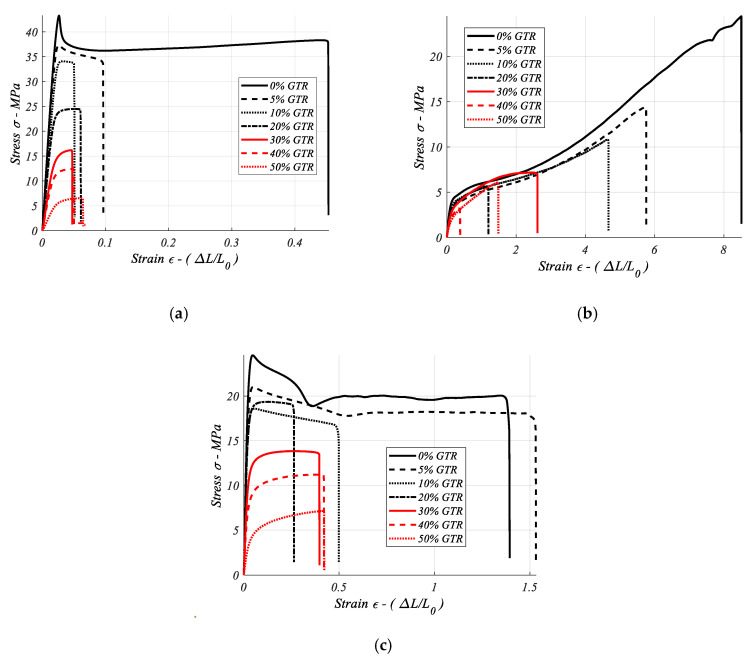
GTR composites curves of stress (σ) vs. strain (ε) tests, for several polymeric composites: (**a**) ABS/GTR; (**b**) EVA/GTR; (**c**) PP/GTR.

Stress-strain figures were obtained from the stress-strain test of EVA, PP, and ABS blended with a range of GTR concentrations, from 0% GTR (neat polymer) to 70% GTR polymeric composites. The stress-strain curves are significantly influenced by GTR additions. For the high end-life tire particle (GTR) concentrations ≥20%, the forms of the curves are concave as is seen in the red curves in Figure 8.

This article reports laboratory stress-strain tests performed on a series of seven polymer materials blended with increasing GTR amounts (0, 5, 10, 20, 40, 50, 70%) to evaluate the resulting composites. Five specimens were tested until failure to obtain the stress-strain curve (σ–ε), under uniaxial tension.

**Figure 9:**
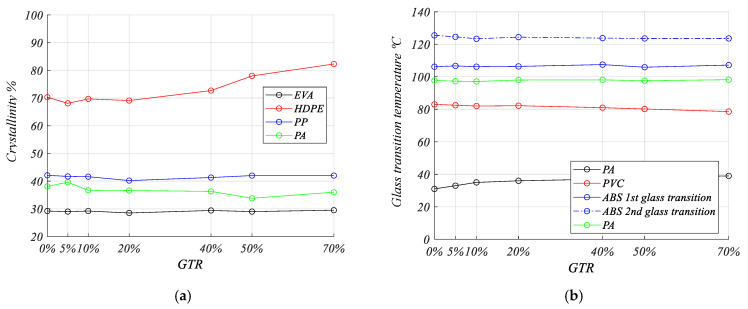
Thermal analysis of end of life tire polymeric blends: (**a**) degree of crystallinity (%) for crystalline polymers and (**b**) glass transition temperature (°C) for amorphous polymers.

A remarkable aspect about the analyzed stress-strain curves (Figure 9) is the greater area under the stress-strain curves for the neat polymer (0%) tested when GTR amounts are adding in the composite matrix the area, and so the σ and ε parameters decrease remarkably, according to the GTR amount added in the polymeric matrix. An explanation for this behavior will be analyzed in the next section on morphology analysis by Scanning Electron Microscopy and is related to the low compatibility between both composite phases: polymer/GTR particles. One interesting aspect is that for specific composites the stress-strain characteristic improves the mechanical behavior, like for instance for EVA + 5% GTR, in which the strain increases slightly compared to the neat polymer.

### 3.9. Thermal Analysis

Differential Scanning Calorimetry (DSC) tests have been performed and thus the crystallinity and glass transition temperature has been obtained for all the neat polymers (0% GTR) and the polymer/GTR composites.

Low relevant changes in the crystallinity (%) for PP, EVA, and PA composites are shown (Figure 9a), but for HDPE increases in the crystallinity are seen, and the amount of GTR in the polyethylene matrix favors the increases of crystallinity in the HDPE matrix. On the other hand the analysis of glass transition temperatures (°C) of the composites (Figure 9b) shows very low changes in the different glass transition temperatures analyzed, so this behavior reveals the low interaction between both phases of the analyzed composites: ground tire rubber and the polymeric matrix.

### 3.10. Morphology Analysis

**Figure 10:**
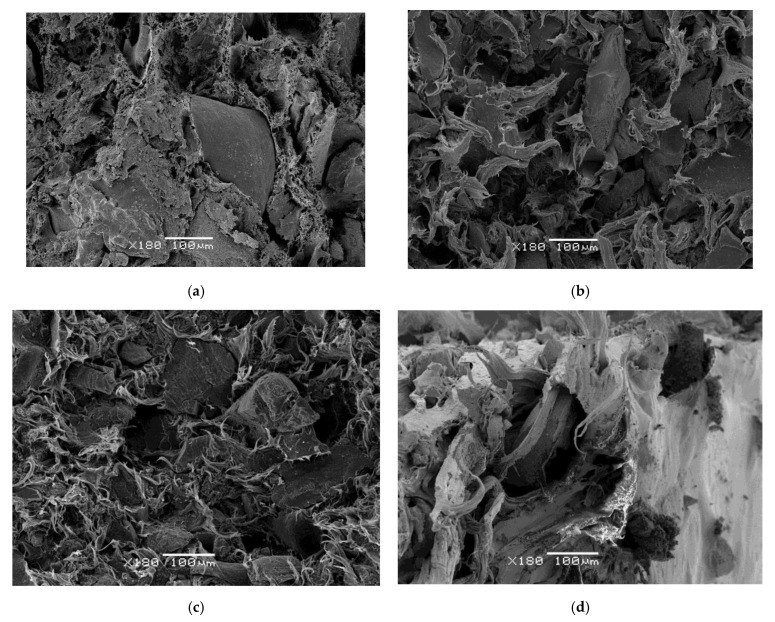

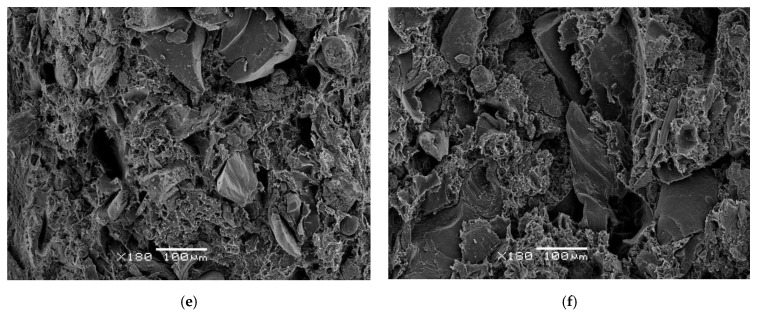
Scanning electron microscopy images, at 180 magnification, of several polymeric composites and different GTR amounts: (**a**) 80% PVC + 20% GTR. (**b**) 50% polypropylene + 50% GTR. (**c**) 30% HDPE + 70% GTR. (**d**) 80% EVA + 20% GTR. (**e**) 60% PS + 40% GTR. (**f**) 50% ABS + 50% GTR.

In Figure 10 scanning electron microscopy images (Jeol, Tokyo, Japan), have been provided. The image analysis reveals some remarkable changes in the interphase composite whereby for a low percent of GTR in the composites, up to 20%, the GTR particle reinforcement shows good integration with the polymeric matrix, as is seen in Figure 10a,d; on the other hand, for higher GTR amounts (40% and 50% of GTR in the composites), Figure 10b,e,f shows integration difficulties with the rest of the polymeric matrix, so the agglomeration of tire microparticles in the thermoplastic matrix affects the material structure and in some cases voids from GTR detached particles are seen, which affects the structural stability of the composite and as a consequence, the mechanical behavior, worsening mechanical properties. Finally, higher GTR amounts in the polymeric matrix (Figure 10c) shows agglomeration of GTR particles, causing voids and fissures in the interphase, showing poor compatibility between both phases, which causes damages to the structure of the composite and finally affects the analyzed mechanical properties.

## 4. Composites Mechanical Behavior Comparison

In Section 3 the results show that the optimum mechanical behavior in polymer/GTR composites is obtained for *p* < 200 μm, with few exceptions. For this reason, this section only considered the lowest particle size: less than 200 μm for the seven polymers with GTR percent, as it is shown in Figure 8.

**Figure 11:**
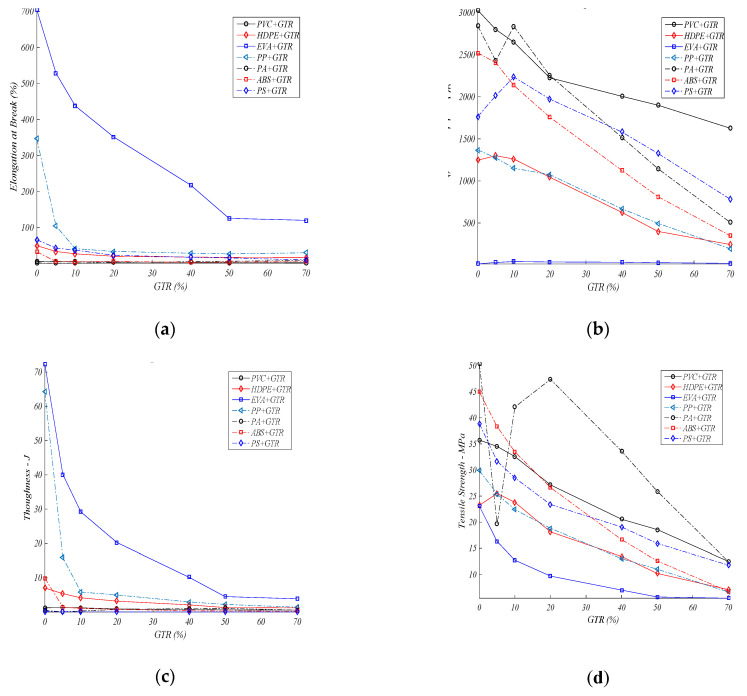
Mechanical properties analysis: (**a**) elongation at break (%), (**b**) Young’s modulus (MPa), (**c**) toughness (J), (**d**) tensile-strength (MPa), for several polymers and GTR amounts (particle diameter lower than 200 μm).

In Figure 11a the elongation at break in polymeric composites is analyzed. For EVA composites we can see the maximum of this specific property, and also with GTR additions in the polymeric matrix, from 704%, 528%, 437%, and finally 351%, for some neat polymers, and GTR amounts: 5% GTR, 10% GTR, and 20% GTR, and these values show the deterioration of the property regarding EVA with no GTR. The elongation property falls remarkably with the incorporation of GTR microparticles, and the rise in elongation suffered after introducing amounts of GTR to PA composites could be considered as an exception. In many cases studied the decrease in elongation at break is caused by poor interfacial adhesion between both components. The deformation feature of the rubber is lower than the deformation capacity of the polymer, which also explains the decreases in elongation and hardness features. Similar behaviors are seen for the toughness (Figure 11c) for EVA + GTR composites. The rest of the polymers give low results for the properties elongation at the break—toughness, which are very important properties in industrial applications. In the Young’s modulus analysis (Figure 11b), EVA, HDPE, and PS composites have optimum behavior at low percentages of GTR (5–10%) addition in the composites. Generally, the addition of larger amounts GTR causes drops in the Young’s modulus. Another exception is EVA, which has low Young’s modulus values (13.26 MPa) for neat polymer, which improve with GTR addition, 41.67 MPa (EVA + 10% GTR). Tensile strength (Figure 8) drops for >10% of GTR amounts, except for the HDPE composites, which behavior improves with the incorporation of GTR particles (5–10%). Regarding the toughness property (Figure 10c), the drops are major and go from 72.3 J for neat EVA to 40 J and 29.2 J (5–10% GTR). Analyzing the toughness (J) graphs, among all pure polymers the greater energy to break are seen for PP (64.22 J) and EVA (72.32 J). With the incorporation of larger GTR amounts the energy at break (J) drops dramatically, while for EVA-PP, after adding 10–20% GTR, the breaking energy of the composites decreases. For ABS-PP-HDPE, the inclusion of larger GTR amounts deteriorates the resistance to breakage (J) and a major drop of resistance to breakage is suffered for a very low percent of GTR (from 5%). Singular cases are the PVC and PA polymers, which have low breaking energy properties for the neat polymer matrix and the toughness improves with the addition of GTR to the polymeric matrix.

### Discussion

Usually, the addition of out-of-use tires particles (GTR) to polymeric composites generally causes a decrease in the four mechanical characteristics analyzed: Young’s modulus, tensile strength, elongation, toughness (Figure 8), but the behavior of these mechanical properties, however, changes according to the GTR particle diameter. For smaller particles (<200 μm), the drop is slightly, and it stabilizes for 20% added GTR, whereas for particles of 200–500 μm, generally, the decreases of the mechanical properties are greater and do not stabilize with increases of the presence of GTR in the composites. The explanation is that small particles, with low presence in the composite matrix, integrate better into the polymeric phase, adding to the composites’ higher features (from particles or matrix), however with for particles with the highest diameters: >500 μm, or with low size particles but high GTR amounts the interface adhesion is poor, and this then affects the structure of the internal composites, which become more fragile, provoking cracks-fractures that reduce and deteriorate the mechanical properties of the GTR composites. It should be noted that, with some exceptions, better behavior of the analyzed mechanical properties is observed for samples with particles of size <200 µm, followed by composites with particles of diameters 200–500 µm, and finally for particles >500 µm.

It is remarkable that the mechanical characteristics are so deeply influenced, evidently, by the GTR characteristics and amounts. The properties of the neat polymer are maintained, between 10–20% of GTR presence (*p* < 200 μm), where the interaction of GTR particles with the matrix is, generally rather weak, and starting from amounts > 20% GTR, the mechanical characteristics drop dramatically.

## 5. Conclusions

One of the conclusions from the present analysis is that particle diameter influences the analyzed mechanical properties, and it has been proved that for low sized GTR particles (<200 μm) the mechanical behavior is better except in the PA/GTR composites, so the analyzed mechanical properties improve with the GTR smallest particle diameter (<200 μm); this is caused by the fact the interfacial adhesion behavior between both phases is better for a minimum particle diameter (*p* < 200 μm) than for the highest particle diameter (*p* > 500 μm). Otherwise, it can be deduced from the comparative study from PA, HDPE, ABS, PVC, EVA, PP, PS with added amounts of GTR, that the analyzed composites’ features change depending on the GTR amount in the polymeric composite, so some properties change according to the GTR amount in the matrix.

Table 3 lists the highest standards of the characteristics than the neat polymer (0% GTR), which we suppose are the optimum and higher value, but we can see in Table 3, that some exceptions are highlighted, so for EVA, PA, PS, HDPE composites some mechanical features improve with the addition of GTR microparticles (diameter *p* < 200 µm) to the matrix of the polymer. Thus, after adding GTR (5–10%) to a matrix and for small size particles, the Young’s modulus in HDPE, PS, EVA composites increases slightly, however other mechanical characteristics decrease (EVA, PS, HDPE composites with GTR). The improvement behavior is caused by the better reinforcement of the matrix for these low GTR concentrations. For GTR amounts over >10%, with all particle diameters, every mechanical feature is reduced; a remarkable exception however is PA composites, the improvements of which with increasing amounts of GTR in the matrix are a remarkable exception which shows its different behavior from the other analyzed polymers that show a dependence on the percentage of GTR, and also with the size of the particles and for higher amounts and particle diameter presence in the composites increases in elongation and toughness are seen. Finally, also remarkable that PP and ABS do not show any improvement for any mechanical property from the neat polymer for any GTR composite analyzed, neither for the particle sizes analyzed, which suggests a deterioration of the internal structure in the presence of GTR in these polymeric matrices.

In Table 3 and Table 4, the highest values of the property mainly correspond to the neat polymer (0% GTR), for EVA, HDPE, PA, but for PS some mechanical properties are improved by the addition of GTR micro-particle (<200 µm) amounts to the matrix of the polymer (Table 3). The mechanical parameters are remarkably influenced by the GTR presence. The stress-strain test data show that the mechanical properties of the neat polymer matrix are maintained between 10–20% of GTR amounts (*p* < 200 μm). Integration of GTR particles in the polymeric matrix is weak and therefore, it accepts this low content of GTR in its matrix. For amounts >20% of GTR in the polymeric matrix, the mechanical features decrease remarkably.

Finally, the analysis of these composites shows that in general a 5–10% GTR concentration is the percentage limit value for acceptable mechanical characteristics. The finding shall allow the use of GTR in some industrial applications, through applications that could be a recycling solution for GTR in the industrial field, and so we could recycle the vast quantities of out of use GTR that represent an environmental problem due to the extreme difficulties of rubber recycling due to its crosslinked structure, Other works [30,31,32,33,34,35,36] present efforts in this direction and the present article pretends to be an effort in the field of characterization of composites with GTR, which could provide some recycling solutions in the tires and rubber fields.

## Figures and Tables

**Table 1:** Technical data of the seven thermoplastics used.

Polymer Type	Commercial Name	Melt Flow Index (g/min)	Density (kg/m^3^)
PVC	Etinox	1.35	1225
EVA	Alcudia PA 539 type	0.20	937
HDPE	Alcudia 4810-B	1.35	960
PA 6	Ultramid B3S	1.55	1130
ABS	Terluran^®^ HH-106	1.45	1050
PP	Isplen^®^ 099 K2M type	0.55	902
PS	Polystyrol 486 M	1.45	1050

**Table 2:** Processing data of the 7 thermoplastics used.

Polymer Type	Processing Temperature (°C)	Melting Temperature (°C)	Pressing Temperature (°C)
PVC	195–200 °C	200 °C	210 °C
EVA	105–110 °C	110 °C	120 °C
HDPE	150–155 °C	155 °C	170 °C
PA 6	195–200 °C	220 °C	210 °C
ABS	180–185 °C	230 °C	195 °C
PP	155–165 °C	165 °C	165 °C
PS	180–185 °C	180°C	195 °C

**Table 3:** Optimum values of analyzed features and GTR composite (*p* < 200 µm).

Composite	Improved Properties in GTR Polymeric Composites (*p* < 200 μm)	
PVC/GTR	Elongation at break: 6.31% (5% GTR)	Toughness: 1.31 J (5% GTR)
PA/GTR	Elongation at break: 8.46% (70% GTR)	Toughness: 1.09 J (50% GTR)
HDPE/GTR	Young’s modulus: 1300.11 MPa (5% GTR)	Tensile strength: 25.51 MPa(5% GTR)
PS/GTR	Young’s modulus: 2235.42 MPa (10% GTR)	
EVA/GTR	Young’s modulus: 41.67 MPa (10% GTR)	

**Table 4:** Values of each neat polymers mechanical properties analyzed without GTR reinforcement.

Polymer Composite	Young’s Modulus (MPa)	Tensile Strength (MPa)	Elongation at Break (%)	Toughness (J)
PVC	3028.89	35.75	5.4	1.25
EVA	13.26	23.08	704.6	72.32
HDPE	1246.34	23.23	50	7
PP	1368.65	29.9	346.71	64.22
ABS	2522.37	44.98	32.91	9.9
PA	2841.47	50.41	2.18	0.45
PS	1764.48	38.89	66.27	0.20
